# Ingestional Toxicity of Radiation-Dependent Metabolites of the Host Plant for the Pale Grass Blue Butterfly: A Mechanism of Field Effects of Radioactive Pollution in Fukushima

**DOI:** 10.3390/life12050615

**Published:** 2022-04-20

**Authors:** Akari Morita, Ko Sakauchi, Wataru Taira, Joji M. Otaki

**Affiliations:** 1 The BCPH Unit of Molecular Physiology, Department of Chemistry, Biology and Marine Science, Faculty of Science, University of the Ryukyus, Okinawa 903-0213, Japan; e183422@eve.u-ryukyu.ac.jp (A.M.); yamatoshijimi@sm1044.skr.u-ryukyu.ac.jp (K.S.); 2 Research Planning Office, University of the Ryukyus, Okinawa 903-0213, Japan; wataira@lab.u-ryukyu.ac.jp

**Keywords:** radioactive pollution, Fukushima nuclear accident, lauric acid, alfuzosin, ikarugamycin, plant secondary metabolite, artificial diet, *Zizeeria maha*, *Oxalis corniculata*, low-dose exposure

## Abstract

Biological effects of the Fukushima nuclear accident have been reported in various organisms, including the pale grass blue butterfly *Zizeeria maha* and its host plant *Oxalis corniculata*. This plant upregulates various secondary metabolites in response to low-dose radiation exposure, which may contribute to the high mortality and abnormality rates of the butterfly in Fukushima. However, this field effect hypothesis has not been experimentally tested. Here, using an artificial diet for larvae, we examined the ingestional toxicity of three radiation-dependent plant metabolites annotated in a previous metabolomic study: lauric acid (a saturated fatty acid), alfuzosin (an adrenergic receptor antagonist), and ikarugamycin (an antibiotic likely from endophytic bacteria). Ingestion of lauric acid or alfuzosin caused a significant decrease in the pupation, eclosion (survival), and normality rates, indicating toxicity of these compounds. Lauric acid made the egg-larval days significantly longer, indicating larval growth retardation. In contrast, ikarugamycin caused a significant increase in the pupation and eclosion rates, probably due to the protection of the diet from fungi and bacteria. These results suggest that at least some of the radiation-dependent plant metabolites, such as lauric acid, contribute to the deleterious effects of radioactive pollution on the butterfly in Fukushima, providing experimental evidence for the field effect hypothesis.

## 1. Introduction

Anthropogenic impacts on wild organisms have been an important scientific and political issue worldwide in this century. Human activities often involve local and global scale pollution of air, water, soil, and ocean, leading to climate changes and human health disorders [1,2]. For example, anthropogenic radionuclides from nuclear bomb tests and nuclear power plant accidents can be found worldwide [3,4,5,6,7]. It is thus important to understand precisely how severely wild organisms are affected by human activities and in what ways. To this end, butterflies have often been used as ecological indicators because of their advantages over other organisms [8,9]. For example, (1) butterflies are often conspicuous and abundant in the field and easy to identify at the species level, (2) a wealth of information on life history is available, (3) rich museum specimens are often available, and (4) many amateur lepidopterists may join field studies covering a wide geographic range. These advantages of using butterflies are invaluable for field studies. Not surprisingly, changes in butterfly species in abundance, range, phenology, and diversity have been used as key factors to understand recent environmental influences in many studies [10,11,12,13,14,15,16]. Occasionally, studies have focused on a single or a few indicator species [17,18,19,20,21,22,23]. An advantage of a single-species approach is to couple field surveys and laboratory experiments to understand what occurs in the field.

The pale grass blue butterfly, *Zizeeria maha*, has been used as a field indicator and laboratory model species to understand evolutionary and developmental plasticity in response to environmental changes [17,24,25,26,27,28]. In this butterfly, an environmentally induced plastic phenotype was genetically assimilated in the laboratory experiment and in the field, which was probably one of the best pieces of evidence for genetic assimilation [29,30]. Just after the establishment of the pale grass blue butterfly as a laboratory model species that can also be used as a field indicator species, the Fukushima nuclear accident occurred in March 2011. Anthropogenic radioactive materials from the Fukushima Dai-ichi Nuclear Power Plant (FDNPP) heavily polluted the east side of Tohoku district in Japan. The northern range margin of the pale grass blue butterfly was located 380 km away from the FDNPP [17], and the polluted area in Fukushima is completely covered by the distribution range of the pale grass blue butterfly. Without question, the pale grass blue butterfly was the logical choice for studying the biological effects of the Fukushima nuclear accident.

The Fukushima nuclear accident was reported to have caused various biological and ecological effects on animals, such as birds [31,32,33], insects [34,35,36,37,38,39], Japanese monkeys [40,41,42], and intertidal invertebrates [43], plants such as rice [44,45], fir trees [46], red pine trees [47], and the creeping wood sorrel *Oxalis corniculata* [48,49,50], and soil microbes [51]. A series of our studies [31,32,33,34,52,53,54,55,56,57,58,59,60,61,62,63] demonstrated that the pale grass blue butterfly has been severely affected by the Fukushima nuclear accident. One of the pieces of important evidence was provided by the internal exposure experiment, in which the contaminated host plant leaves collected from Fukushima were given to larvae from Okinawa (where radioactive contamination is minimal), resulting in high mortality and abnormality rates. However, when an artificial diet containing pure radioactive cesium (^137^Cs) was given to larvae, no change in the survival rate was observed [64]. A similar discrepancy between field and laboratory results has been observed in the case of the Chernobyl nuclear accident [65,66]. This field-laboratory paradox was explained by the field effect hypothesis: the host plant in the field responded to low-level radiation stress by upregulating metabolites that were toxic to larvae as a part of plant defense mechanisms [59,67]. Subsequent studies have reported upregulated and downregulated metabolites and nutrients in plant leaves [48,49,50], supporting this field effect hypothesis.

In a previous metabolomic study [49], the creeping wood sorrel in Okinawa was irradiated by contaminated soil collected from Fukushima, and the leaf samples (the edible part for larvae) were subjected to GC–MS (gas chromatography–mass spectrometry) and LC–MS (liquid chromatography–mass spectrometry) analyses. Under the acute low-dose radiation conditions, 5.7 mGy (34 μGy/h for seven days), many peaks were significantly upregulated, although most of them were annotated as multiple compounds or not annotated at all. One of the upregulated peaks was singularly annotated as lauric acid by targeted GC–MS analysis, and two of the upregulated peaks were singularly annotated as alfuzosin and ikarugamycin by LC–MS analysis. Therefore, the potential toxic effects of these three compounds are of great interest.

Lauric acid is a saturated fatty acid, also called dodecanoic acid, that can be found widely in plants. Lauric acid shows a wide variety of bioactivities as a plant defense, volatile against *Staphylococcus* [68,69], *Mycobacterium tuberculosis* [70], fungus [71], and *Phytophthora sojae*, an agriculturally important plant pathogen that belongs to Protista [72]. Notably, extracts from *Vitex* species containing lauric acid have larvicidal activity against a mosquito species, *Culex quinquefasciatus* [73]. Lauric acid is likely sensed at least by an insect, *Holotrichia parallela* [74], as an odorant. Accordingly, it is reasonable to hypothesize a larvicidal activity of lauric acid in *O. corniculata* against larvae of the pale grass blue butterfly.

Alfuzosin is a synthetic α_1_-adrenergic receptor antagonist used widely for the treatment of benign prostatic hyperplasia [75,76,77,78]. Because it is synthetic, it is unlikely to be present naturally in the plant. However, because the LC–MS peak annotated as alfuzosin has very similar (virtually identical) elution time and exact mass with alfuzosin, examination of MS/MS (mass spectrometry/mass spectrometry, i.e., tandem mass spectrometry) spectrograms was required to differentiate alfuzosin and this unknown plant metabolite (called the alfuzosin-related compound hereafter) [49]. Faced with the fact that the exact identity of the alfuzosin-related compound cannot be determined easily, we tested the toxicity of alfuzosin itself in this study, assuming that alfuzosin and its related metabolite may have similar biological effects on larvae of the pale grass blue butterfly.

Ikarugamycin is an antiprotozoal agent isolated originally from the soil bacterium *Streptomyces phaeochromogenes* var. *ikaruganensis* [79]. Importantly, ikarugamycin has also been detected from an endophytic actinomycete, *Streptomyces harbinensis*, from soybean root [80]. Endophytic bacteria have been widely observed in plants [81,82,83,84,85,86], including *O. corniculata* [87,88]. Accordingly, ikarugamycin detected from leaves of *O. corniculata* is likely from an endophytic *Streptomyces* sp. in leaves or roots, which responded to low-level radiation [49,50]. Ikarugamycin and its derivatives are antifungal [89] and antibacterial [89,90] agents and inhibit clathrin-mediated endocytosis in eukaryotic cell lines [91].

These three upregulated metabolites have been hypothesized to function as toxicants for larvae of the pale grass blue butterfly under low-level radiation stress. In other words, these compounds are candidate causal substances for the ecological field effects of low-level radiation pollution in Fukushima. In this study, we tested the above hypothesis by investigating the ingestional toxicity of these compounds using a novel artificial diet that has a reduced leaf content of *O. corniculata*.

To detect their potential toxicity, we examined three aspects of development: metamorphosis rates, developmental periods, and adult wing size. The metamorphosis rates were used to detect the number of surviving individuals after metamorphosis and included the following: the pupation rate, the eclosion (survival) rate, and the normality rate. The developmental periods were used to detect developmental retardation or acceleration and included the following: the egg-larval period, the pupal period, and the immature period. Adult wing size included both male and female forewing sizes. In this way, we examined high mortality and abnormalities, growth retardation, and smaller forewing size, which have been detected as biological effects of the Fukushima nuclear accident in previous studies [34,35,36,37].

## 2. Materials and Methods

### 2.1. Egg Collection and Larval Rearing

Egg collection and rearing were performed according to Hiyama et al. (2010) [24] and other related publications [34,52,56] with some minor modifications, as described briefly below. Adults of the pale grass blue butterfly *Z. maha* and its host plant, the creeping wood sorrel *O. corniculata*, were collected at the University of the Ryukyus and its vicinity. The whole plant was placed in a pot and set in a glass tank (300 mm × 300 mm × 300 mm) in which approximately three female butterflies and a few male butterflies were confined at a time. A single trial of egg collection was performed for a period of four days. All rearing processes were executed under the conditions where light was automatically turned on from 6:00 a.m. to 10:00 p.m. (L16:D8) and room temperature was set at 27 °C.

After eggs were deposited on the leaves of the host plant, the plant pot was removed from the glass tank and covered with a plastic bag. When the leaves were eaten enough by newly hatched larvae, they were transferred to a transparent plastic container (150 mm × 150 mm × 55 mm), to which a new bunch of the host plant leaves was supplied every day. Larvae were reared with fresh leaves for 14 days from the beginning of egg collection. Larvae were then randomly divided into different treatment groups: a fresh leaf group, an artificial diet group with no test additive (0 mg/g), and a few artificial diet groups with different concentrations of a test additive (0.01 mg/g, 0.1 mg/g, and 1 mg/g). One group was reared in one container that housed 15–25 larvae, depending on the total number of larvae that were obtained simultaneously from a single egg collection trial. The larvae obtained from a single trial were all siblings; they constitute a sibling group. In this way, genetic bias was minimized. The artificial diet was given as four small square lumps (10 mm × 8 mm × 3 mm per lump) at four corners in a container.

We cleaned containers and changed old lumps of the artificial diet for fresh ones every day, but unexpected deaths that were apparently unrelated to the toxicity of the test additives could not be entirely avoided. This is probably partly because we use fresh leaves collected from the field without sterilization because the sterilization process may breakdown or vaporize some ingredients in fresh leaves important for larvae to initiate eating behavior (such as oxalic acid as an eating initiator [92]). Pupation and eclosion were checked every morning, roughly from 8:30 am to 1:00 pm, so that the data on the developmental periods (days) could be obtained later. We set a criterion that the eclosion (survival) rate of the sibling group of the artificial diet without a test additive should be more than 45% to be considered a successful rearing trial. Sibling groups with eclosion rates below this criterion were considered technical failures and excluded from subsequent analyses. This threshold was set as the gap based on our rearing experience. After pupation, pupae inside the container were transferred to small petri dishes individually. Soon after eclosion, adult butterflies were frozen until subsequent analyses.

### 2.2. Artificial Diet Preparation

Larvae require leaves (or some plant chemicals) as a component of an artificial diet to eat, but the leaf content in an artificial diet should be minimized to test toxicological effects of a metabolite in leaves themselves. Furthermore, the process of collecting fresh leaves is the most laborious and time-consuming process for preparing an artificial diet. To meet these demands, we developed a novel artificial diet for the pale grass blue butterfly for the feeding experiments in this study. We used a commercially available diet, Silk Mate L4M (Nosan Corporation, Yokohama, Kanagawa, Japan), for rearing silkworm. This diet was supplied as powder containing defatted soybean, starch, sugar, cellulose, formative agent, citric acid, mulberry leaf powder, vitamins, minerals, preservative, and antibiotics (diet additives), according to the manufacturer’s specification. For the current study, fresh leaves of the creeping wood sorrel, Silk Mate L4M, and deionized water were mixed at a weight ratio of 1:3:5. Thus, this new diet was named AD-FSW-135.

A possibility that Silk Mate L4M contains lauric acid, the alfuzosin-related compound, and ikarugamycin from mulberry leaves was not considered in this study based on the following reasons, although it cannot be excluded completely. First, ikarugamycin and alfuzosin (and thus the alfuzosin-related compound) have never been reported from mulberry leaves to the best of our knowledge. Besides, their concentrations in the creeping wood sorrel were low (see below). Second, lauric acid is known to be contained in mulberry leaves [93,94]. However, because lauric acid is volatile, it may be minimized during an autoclave sterilization process of Silk Mate L4M. The basal levels of the three compounds that were carried from the fresh leaves in AD-FSW-135 (prepared in this study) were shown to be low (see Section 4).

### 2.3. Lauric Acid, Alfuzosin, and Ikarugamycin

Lauric acid (catalog No. 042-23281, Wako Special Grade; FUJIFILM Wako Chemicals, Tokyo, Japan), alfuzosin (catalog No. PHR1638, Pharmaceutical Secondary Standard, Certified Reference Material; Sigma–Aldrich, St. Louis, MO, USA), and ikarugamycin (catalog No. 15386; Cayman Chemical, Ann Arbor, MI, USA) were purchased. They were in powder form and were added to the diet preparation directly. They were then mixed well manually or using an electric mixer until the diet preparation was visually judged to be homogeneous. In this way, we assumed that the test additives were incorporated evenly into the diet and became solubilized. However, they might not have been solubilized completely at higher concentrations (see Section 4). Concentrations of these test additives were expressed in milligrams per gram of artificial diet (mg/g) throughout this paper. For lauric acid, we tested 0 mg/g (control), 0.01 mg/g, 0.1 mg/g, and 1 mg/g. This range covered the estimated concentration of lauric acid in leaves (see below). For alfuzosin, we tested 0 mg/g (control), 0.01 mg/g, and 0.1 mg/g. For ikarugamycin, we tested 0 mg/g (control) and 0.01 mg/g. These ranges of alfuzosin and ikarugamycin were much greater than the concentrations of the alfuzosin-related compound and ikarugamycin in leaves (see below). Nevertheless, we tested these concentrations because excessive doses are often necessary to obtain the median toxic dose TD_50_ and the median lethal dose LD_50_ and because comparison among the three compounds at the same doses may provide us with valuable information on diverse effects of leaf compounds. Basal levels of the compounds from leaves in AD-FSW-135 were not taken into account for analyses due to uncertainty of the estimated leaf concentrations.

### 2.4. Concentration of Lauric Acid in Leaves

**Figure A1:**
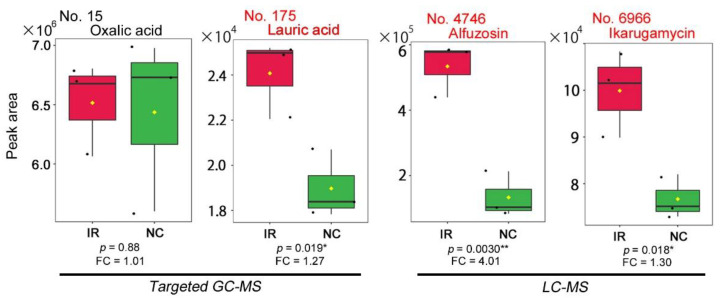
Peak area values of oxalic acid and lauric acid in the targeted GC–MS analysis [49] (left) and peak area values of alfuzosin-related compound and ikarugamycin in the LC–MS analysis [49] (right). Box plots for lauric acid, alfuzosin, and ikarugamycin are also shown in Sakauchi et al. (2021) [49]. IR (shown in red) and NC (shown in green) indicate irradiated samples (*n* = 3) and nonirradiated control samples (*n* = 3), respectively. A single black dot represents the mean value of triplicate of a sample. FC indicates fold change of mean values from nonirradiated to irradiated samples. These plots were produced using MetaboAnalyst [96]. Asterisks indicate levels of statistical significance; *, *p* < 0.05; **, *p* < 0.01 (*t*-test).

The concentration of lauric acid in leaves was roughly estimated as follows. Lauric acid in leaves was discovered by the targeted method of GC–MS [49] (Appendix A). Because this metabolite was targeted based on previously known chemical information, the identification and peak area data were more credible than the nontargeted method. Although each compound has a different detection efficiency in GC–MS, it is possible to roughly compare peak area values among targeted metabolites detected simultaneously from the same samples. One of the targeted metabolites was oxalic acid. The concentration of oxalic acid in leaves of *O. corniculata* has been reported to be 16.9 mg/g (leaf) [95]. On the other hand, the mean peak area value of oxalic acid (No. 15) in nonirradiated samples in the targeted GC–MS analysis was 6,409,017 (*n* = 3) (Figure A1) [49]. Similarly, the mean peak area value of lauric acid (No. 175) in nonirradiated samples in the targeted GC–MS analysis was 18,899 (*n* = 3) (Figure A1) [49]. Therefore, the concentration of lauric acid in nonirradiated leaves was calculated to be 0.050 mg/g (leaf). When irradiated, the mean peak area value of lauric acid was 23,977 (*n* = 3) [49], and it increased approximately 1.27 times to 0.063 mg/g (leaf) under the previous experimental conditions [49].

### 2.5. Concentration of the Alfuzosin-Related Compound in Leaves

**Figure A2:**
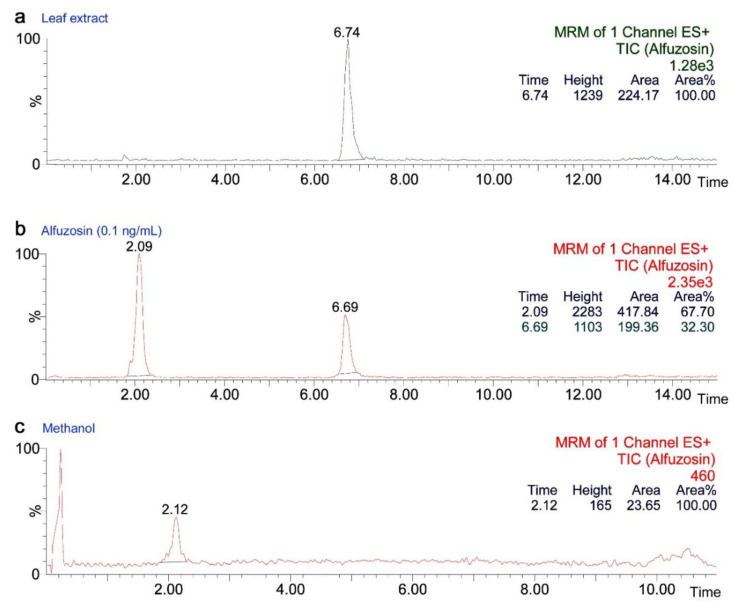
Identification of the alfuzosin-related metabolite by HPLC. (**a**) Leaf extract. A peak at 6.74 min was observed. This is one of the triplicate results. The peak area value here is 224.17. (**b**) Alfuzosin standard (Sigma–Aldrich) 0.1 ng/mL in methanol. The peak at 6.69 min is attributed to alfuzosin, and the peak at 2.09 min is attributed to an impurity in methanol. The peak area value of alfuzosin, 199.36, is similar to that of the alfuzosin-related compound. (**c**) Methanol only. A peak at 2.12 min was observed, demonstrating impurity.

The concentration of the unknown alfuzosin-related compound in *Oxalis* leaves was estimated by HPLC spectrograms of LC–MS newly performed in this study (Figure A2). Leaf samples for the previous study (Figure A1) [49] and the current study (Figure A2) were identical. Sample preparation procedures followed a previous LC–MS study [49]. Washed fresh leaves of *O. corniculata* (100 mg) were frozen, ground, and thoroughly homogenized with methanol (300 μL). After a brief centrifugation, 200 μL was recovered, from which 10 μL was subjected to LC–MS analysis. This extraction process can be considered a total volume increase to 400 μL, assuming that leaf density is close to that of water (1.0 g/mL). The experimental conditions for the LC–MS analysis in the present study are described in Appendix A.

The alfuzosin-related compound in the leaf extract had a mean peak area value of 201.81 in triplicate of a sample (Figure A2a). This peak area value was similar to that of the alfuzosin standard (Sigma–Aldrich), 199.36, when 0.10 ng/mL methanol extract was analyzed (Figure A2b). Although the alfuzosin standard spectrum showed an additional peak, this peak was found to be an impurity peak in methanol (Figure A2c). Considering the volume conversion factor, ×4.0, the concentration of the alfuzosin-related compound in nonirradiated leaves was estimated to be 0.40 ng/g (leaf). In a previous metabolomic study, the mean peak area values of the alfuzosin-related compound (No. 4746) were 133,169 (*n* = 3) (without irradiation) and 534,069 (*n* = 3) (irradiated) (Figure A1) [49]. Thus, when irradiated, the area value increased 4.01 times to 1.6 ng/mg (leaf) under the previous experimental conditions.

### 2.6. Concentration of Ikarugamycin in Leaves

The concentration of ikarugamycin in leaves was estimated based on the previous peak area values of LC–MS (Figure A1) [49]. Alfuzosin-related compound (No. 4746) had a mean peak area value of 133,169 (*n* = 3) in nonirradiated samples, whereas ikarugamycin had a peak area value of 76,713 (*n* = 3) from the same samples [49]. Assuming that it is possible to roughly compare peak area values among metabolites detected simultaneously from the same samples, the concentration of ikarugamycin was calculated to be 0.20 ng/g (leaf) based on the estimated concentration of the alfuzosin-related compound in leaves. When irradiated, the mean peak area value of ikarugamycin was 99,905. Thus, the area value increased 1.30 times to 0.26 ng/g (leaf) under the previous experimental conditions.

### 2.7. Toxicological Output Data

To understand the toxicity of the three metabolites, we recorded three metamorphosis-related data as the number of individuals as follows: the number of individuals that successfully pupated (the number of pupae), the number of individuals that successfully eclosed (the number of adults), and the number of individuals that successfully eclosed without wrinkled wings (the number of normal adults), as shown in Appendix B (Table A1, Table A2, Table A3 and Table A4). These three numbers were used for calculating the three metamorphosis rates: the pupation rate, the eclosion rate, and the normality rate. For calculations, these numbers were divided by the starting number of larvae, and the results were expressed as a percentage. The eclosion rate was also called the “survival rate”.

We also recorded three developmental period data as the number of days: the number of days from the time point when egg collection started to pupation (the egg-larval days), the number of days from pupation to eclosion (the papal days), and the number of days from the time point when egg collection started to eclosion (the immature days). The immature days are simple summation of the egg-larval days and the pupal days. The egg-larval days included prepupal days.

Additionally, we measured adult forewing size from the wing base to the apex using a desktop digital microscope SKM-2000 with its associated software SK-measure (Saito Kogaku, Yokohama, Kanagawa, Japan). Because male and female forewing sizes are known to be different in this species [24,64], forewing size data were compiled sex-dependently. Individuals with wrinkled wings were not subjected to size measurements. The developmental period data and the forewing size data were compiled in Table S1. The numbers of individuals in repeated biological trials were also shown in Table S1.

### 2.8. Statistical Analysis

A treatment group (0.01 mg/g, 0.1 mg/g, or 1 mg/g of a test additive of interest) was statistically compared to a corresponding no treatment (control) group (0 mg/g). We performed the χ^2^ test for the data on the number of individuals that produced the pupation rate, the eclosion rate, and the normality rate. The χ^2^ test was also performed for evaluating the performance of the artificial diets and for comparing the normalized eclosion and normality rates between lauric acid and alfuzosin. Yates’ correction was not performed. We performed either Student’s *t*-test (equal variance) or Welch’s *t*-test (unequal variance) for the egg-larval days, the pupal days, the immature days, and the forewing size, assuming that they were normally distributed. Bonferroni or other correction was not performed. Statistical analyses were performed using Microsoft Excel (Office 365), JSTAT (Yokohama, Kanagawa, Japan), and MetaboAnalyst [96]. MetaboAnalyst was also used to produce box plots of metabolites obtained in a previous study [49].

## 3. Results

### 3.1. Performance of the Artificial Diet AD-FSW-135

**Figure 1:**
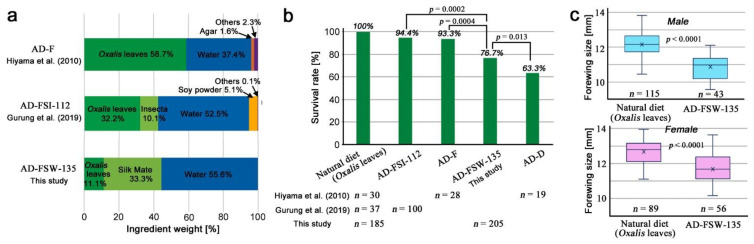
Ingredients and performance of artificial diets. (**a**) Ingredients and their weight percentages. AD-FSW-135 has a reduced leaf content. It also has simplified contents with just three ingredients. (**b**) Survival rate (eclosion rate). AD-FSW-135 shows a higher survival rate than AD-D [24] and a lower rate than AD-F [24] and AD-FSI-112 [64]. The *p*-values obtained from the χ^2^ test are shown. The AD-FSW-135 results were obtained from ten biological repeats (see Supplementary Table S1). (**c**) Male (top) and female (bottom) forewing size. The *p*-values obtained from the *t*-test between the natural diet and AD-FSW-135 are indicated. Both natural diet and AD-FSW-135 results were obtained from ten biological repeats (see Supplementary Table S1).

The new artificial diet AD-FSW-135 developed for this study was compared with the previous diets, AD-F (artificial diet with fresh leaves) [24] and AD-FSI-112 (artificial diet with fresh leaves, soy powder, and Insecta F-II (Nosan Corporation)) [64], in terms of ingredients (Figure 1a). The most important difference among the three artificial diets was the leaf content. *Oxalis* leaves occupied 58.7% of the diet in AD-F [24] and 32.2% in AF-FSI-112 [64]. In contrast, *Oxalis* leaves occupied only 11.1% in the new diet AD-FSW-135. In other words, AD-FSW-135 (this study) contained approximately one-fifth and one-third of fresh leaves of the previous diets AD-F [24] and AD-FSI-112 [64], respectively. The reduced leaf content in the new diet AD-FSW-135 was considered important for toxicological tests (see Section 2). This leaf-content reduction was achieved by the introduction of Silk Mate L4M (Nosan Corporation), a commercially available artificial diet for silkworms. In a previous diet, AD-FSI-112, Insecta F-II from the same manufacturer was used [64]. Simplification of the contents for quick and easy preparation with just three ingredients was also an important advantage of the new artificial diet AD-FSW-135 developed for this study.

Throughout the rearing experiments with the new artificial diet AD-FSW-135 containing a compound of interest (called a test additive), we always simultaneously reared a group of larvae with natural fresh leaves of the creeping wood sorrel and another group of larvae with AD-FSW-135 without a test additive (0 mg/g) (Appendix B; Table A1). To evaluate the performance of the new diet AD-FSW-135, the survival (eclosion) rates of larvae without an additive were compared among the previous and present diets (Figure 1b). AD-FSW-135 (this study) had a significantly higher survival rate than AD-D (artificial diet with dried leaves) [24] and a significantly lower rate than AD-FSI-112 [64] and AD-F [24], indicating that the survival rate of AD-FSW-135 (this study) was not very high but was not very low. AD-FSW-135 (this study) was thus considered acceptable for toxicological tests, as long as the majority of larvae could eat AD-FSW-135 and grow.

The forewing size of adult individuals reared with the new diet AD-FSW-135 was reduced in comparison to that of fresh plant leaves (Figure 1c). Males from the natural diet and AD-FSW-135 groups showed forewing sizes of 12.16 ± 0.71 mm (mean ± standard deviation) and 10.88 ± 0.65 mm, respectively. Females from the natural diet and AD-FSW-135 groups showed forewing sizes of 12.67 ± 0.68 mm and 11.68 ± 0.92 mm, respectively. In both sexes, the forewing size was reduced significantly. However, these results were essentially similar to those of the previous diets in terms of size distributions [24,64]; this level of size reduction seems to be inherent in rearing butterflies in artificial diets. Therefore, the forewing size reduction in the AD-FSW-135 results was considered acceptable for toxicological tests in this study.

### 3.2. Lauric Acid

We prepared three concentrations of lauric acid in the artificial diet: 0.01 mg/g, 0.1 mg/g, and 1 mg/g, in addition to the diet without it (0 mg/g). These concentrations covered an estimated concentration of lauric acid in the irradiated leaves of *O. corniculata* of 0.063 mg/g. The number of pupae, eclosion, and normal adults were recorded (Appendix B; Table A2). We examined the toxicity of lauric acid from three different viewpoints: metamorphosis rates (the pupation rate, eclosion rate, and normality rate), developmental periods (egg-larval days, pupal days, and immature days), and adult forewing size.

**Figure 2:**
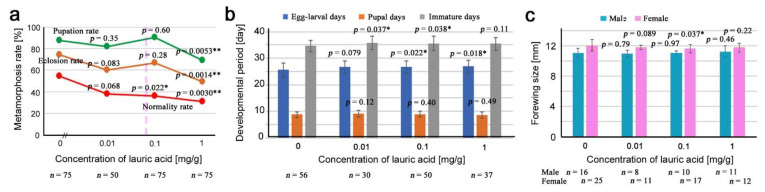
Results of the toxicity test for lauric acid. Asterisks indicate levels of statistical significance in comparison to the control (0 mg/g); *, *p* < 0.05; **, *p* < 0.01. These results were obtained from four biological repeats (see Supplementary Table S1). (**a**) Pupation rate (green), eclosion rate (brown), and normality rate (red). The *p*-values obtained from the χ^2^ test are indicated. The pink vertical broken line indicates a rough position of the estimated concentration of lauric acid in irradiated leaves, 0.063 mg/g. (**b**) Egg-larval days (blue), pupal days (brown), and immature days (gray). The mean values (±standard deviation) are shown as bar height. The *p*-values obtained from the *t*-test are indicated. (**c**) Male (blue green) and female (pink) forewing size. The mean values (±standard deviation) are shown as bar height. The *p*-values obtained from the *t*-test are indicated.

The normality rate linearly decreased in response to the concentration of lauric acid (Figure 2a). The pupation rate and the eclosion rate were largely similar to the normality rate except at 0.1 mg/g, which showed an increase (Figure 2a). In comparison to the diet without lauric acid (0 mg/g), the diet with 1 mg/g showed a significant decrease in the pupation rate, eclosion rate, and normality rate, indicating the toxicity of lauric acid. At 0.1 mg/g and 1 mg/g, the egg-larval days appeared to be significantly longer than the control (0 mg/g) (Figure 2b). The immature days of the 0.01 mg/g and 0.1 mg/g treatments were significantly longer than those of the control (0 mg/g). These results indicate that developmental retardation occurred in the larval periods at all three concentrations of lauric acid. The forewing size of females at 0.1 mg/g was reduced significantly in comparison to the control (0 mg/g), although such a reduction was not observed at other concentrations (Figure 2c).

### 3.3. Alfuzosin

We prepared two concentrations of alfuzosin in the artificial diet, 0.01 mg/g and 0.1 mg/g, in addition to the diet without it (0 mg/g), to compare the results with those of lauric acid, although the lowest concentration used in the present study, 0.01 mg/g, was 6.3 × 10^3^ times higher than an estimated concentration of the alfuzosin-related compound in irradiated leaves, 1.6 ng/g. As in the case of lauric acid, we examined the metamorphosis rates, developmental periods, and adult forewing size (Appendix B; Table A3).

**Figure 3:**
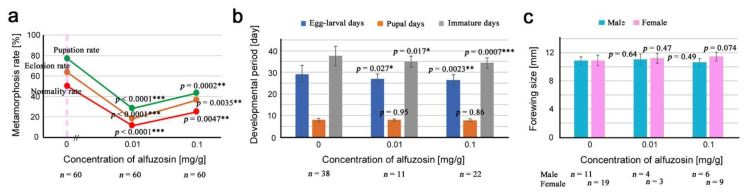
Results of the toxicity test for alfuzosin. Asterisks indicate levels of statistical significance in comparison to the control (0 mg/g); *, *p* < 0.05; **, *p* < 0.01; ***, *p* < 0.001. These results were obtained from three biological repeats (see Supplementary Table S1). (**a**) Pupation rate (green), eclosion rate (brown), and normality rate (red). The *p*-values obtained from the χ^2^ test are indicated. The pink vertical broken line indicates a rough position of the estimated concentration of the alfuzosin-related compound in irradiated leaves, 1.6 ng/g. (**b**) Egg-larval days (blue), pupal days (brown), and immature days (gray). The mean values (±standard deviation) are shown as bar height. The *p*-values obtained from the *t*-test are indicated. (**c**) Male (blue green) and female (pink) forewing size. The mean values (±standard deviation) are shown as bar height. The *p*-values obtained from the *t*-test are indicated.

The pupation rate, eclosion rate, and normality rate all decreased significantly in response to alfuzosin, but not linearly (Figure 3a). Reasons for lower rates at 0.01 mg/g than those at 0.1 mg/g were uncertain but may be technical (see Section 4). The egg-larval days and the immature days were significantly different from those without alfuzosin (0 mg/g) (Figure 3b). Somewhat surprisingly, these differences showed developmental acceleration instead of retardation. The forewing size did not differ from that of the control (0 mg/g), but at 0.1 mg/g in females, the forewing size tended to increase, although the increase was not statistically significant (Figure 3c).

### 3.4. Ikarugamycin

We prepared one concentration of ikarugamycin in the artificial diet, 0.01 mg/g, in addition to the diet without it (0 mg/g) to compare the results with those of lauric acid, although the lowest concentration used in the present study, 0.01 mg/g, was 3.8 × 10^4^ times higher than an estimated concentration of ikarugamycin in irradiated leaves, 0.26 ng/g. As in the cases of lauric acid and alfuzosin, we examined the metamorphosis rates, developmental periods, and adult forewing size (Appendix B; Table A4).

**Figure 4:**
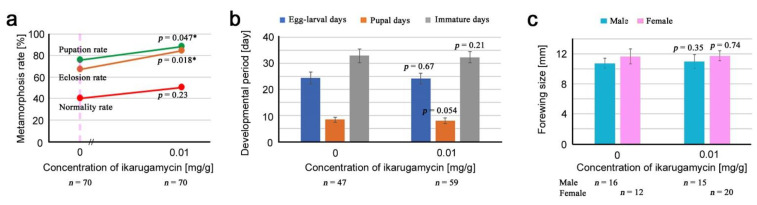
Results of the toxicity test for ikarugamycin. Asterisks indicate levels of statistical significance in comparison to the control (0 mg/g); *, *p* < 0.05. These results were obtained from three biological repeats (see Supplementary Table S1). (**a**) Pupation rate (green), eclosion rate (brown), and normality rate (red). The *p*-values obtained from the χ^2^ test are indicated. The pink vertical broken line indicates a rough position of the estimated concentration of ikarugamycin in irradiated leaves, 0.26 ng/g. (**b**) Egg-larval days (blue), pupal days (brown), and immature days (gray). The mean values (±standard deviation) are shown as bar height. The *p*-values obtained from the *t*-test are indicated. (**c**) Male (blue green) and female (pink) forewing size. The mean values (±standard deviation) are shown as bar height. The *p*-values obtained from the *t*-test are indicated.

Surprisingly, the pupation rate and the eclosion rate increased significantly in response to ikarugamycin, although an increase in the normality rate was not significant (Figure 4a). These results indicate mild drug efficacy of ikarugamycin instead of toxicity. In contrast to lauric acid and alfuzosin, the egg-larval days, pupal days, and immature days at 0.01 mg/g were not different from those without ikarugamycin (0 mg/g) (Figure 4b). The forewing size at 0.01 mg/g did not differ from those without ikarugamycin (0 mg/g) (Figure 4c).

### 3.5. Comparison of Three Compounds

**Figure 5:**
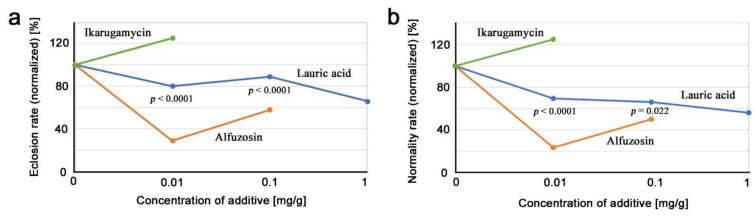
Comparison of the results of the three compounds. The *p*-values obtained from the χ^2^ test between lauric acid and alfuzosin are shown. (**a**) Eclosion (survival) rate (normalized). (**b**) Normality rate (normalized).

Here, we compared the results of the three compounds tested above. The eclosion (survival) rates and the normality rates were normalized so that they became 100% when no compound was added to the diet (0 mg/g) (Appendix B; Table A5 and Table A6) as shown in Figure 5. The eclosion rates (Figure 5a) and the normality rates (Figure 5b) were not very different, but lauric acid exhibited a smooth and gradual dose-dependent decrease in the normality rate curve, although not in the eclosion rate curve, as seen previously (Figure 2a). It is remarkable that the normality rate curves of the three compounds showed different behaviors; in response to concentration, the lauric acid curves decreased dose-dependently, the alfuzosin curves decreased more sharply and not linearly, and the ikarugamycin curves increased (Figure 5b). At the concentration of 0.01 mg/g, where three compounds were able to be compared, the normality rates of lauric acid, alfuzosin, and ikarugamycin were 69.5%, 23.4%, and 125.0%, respectively (Appendix B; Table A6). The differences between lauric acid and alfuzosin appeared to be more significant at the concentration of 0.01 mg/g than 0.1 mg/g in both the eclosion and normality rates, but this may be because of a low solubility of alfuzosin at 0.1 mg/g (see Section 2 and Section 4).

As a convention of toxicological analysis, a regression line was determined using the normality rates as *y* = −24.9*x* + 79.8 (*R*^2^ = 0.40) for lauric acid, and at the normality rate of 50% (*y* = 50), lauric acid concentration *x* was determined as 1.2 mg/g. This is considered equivalent to the median toxic dose, TD_50_. Similarly, a regression line was determined as *y* = −185*x* + 64.6 (*R*^2^ = 0.069) for alfuzosin, and at the normality rate of 50% (*y* = 50), the alfuzosin concentration *x* was determined to be 0.079 mg/g, which is 15 times smaller than that of lauric acid. Just to be sure, if the value at 0.1 mg/g in alfuzosin was erroneously high due to technical reasons, such as low solubility (see Section 4), the LD_50_ value of alfuzosin should be much lower.

Likewise, using the eclosion (survival) rates, a regression line was determined as *y* = −24.8*x* + 90.8 (*R*^2^ = 0.69) for lauric acid, and at the normality rate of 50% (*y* = 50), lauric acid concentration *x* was determined as 1.6 mg/g. This is considered equivalent to the median lethal dose, LD_50_. Similarly, a regression line was determined as *y* = −126*x* + 66.9 (*R*^2^ = 0.038) for alfuzosin, and at the normality rate of 50% (*y* = 50), the alfuzosin concentration *x* was determined as 0.13 mg/g, which is 12 times smaller than that of lauric acid. As in the case of TD_50_, the LD_50_ value of alfuzosin should be much lower if the value at 0.1 mg/g was technically erroneous.

## 4. Discussion

We tested the ingestional toxicity of three compounds, namely, lauric acid, alfuzosin, and ikarugamycin, which were significantly upregulated in *O. corniculata* and annotated by a previous metabolomic study [49]. For convenience, concentration data are compiled in Table 1. In this study, we employed a new artificial diet, AD-FSW-135, which contained a relatively small amount of host plant leaves, occupying just 11% of the entire diet. It is important to keep the leaf content as low as possible in the artificial diet due to an experimental addition of a testing compound. Indeed, the basal levels of the three compounds in the new diet AD-FSW-135 (Table 1) were considered low enough for the current study. This new diet showed acceptable performance based on the survival rate and forewing size, two indexes to evaluate artificial diets [64]. Thus, we believe that the use of AD-FSW-135 in the present study is justifiable but that there is still much room for further improvement of artificial diets.

With this new diet AD-FSW-135, we demonstrated that lauric acid was toxic to larvae dose-dependently in terms of metamorphosis rates, although the larval response was mild. Lauric acid is present in leaves without radiation, and larvae are certainly tolerant to lauric acid at the leaf level of 0.050 mg/g, explaining the gradual dose–response curves. The mild toxicity of lauric acid is expressed in its TD_50_, 1.2 mg/g, in contrast to the TD_50_ of alfuzosin, 0.079 mg/g. LD_50_ values also indicated such a relationship. We observed some toxicity even at the level of 0.01 mg/g, but this may be because larvae were exposed to a sudden rise in lauric acid concentration when the artificial diet was first given. In addition to the changes in the metamorphosis rates, growth retardation was detected at the egg-larval period in response to lauric acid. Furthermore, the forewing size reduction was observed, although only at 0.1 mg/g in females. These results indicate the toxicity of lauric acid on the developmental physiology of the butterfly and appear to be biologically significant in the field because the estimated concentration of lauric acid in irradiated leaves, 0.063 mg/g, was covered by the current study.

In a previous study, the fold change values in the upregulation of lauric acid was 1.27 at low-level radiation exposure; the cumulative dose to the plant was 5.7 mGy (34 μSv/h in a period of seven days) [49]. It is somewhat surprising that the plant significantly responded to this low-level exposure, and we expect that the fold change value may increase further in response to higher levels of radiation exposure. According to Nohara et al. (2014) [52], the ground radiation dose rate in Iitate was 18.9 μSv/h, which is indeed lower than the experimental dose rate used in our study, 34 μSv/h. However, experimental irradiation in the present study was only by external exposure during a very limited period of time (seven days), but in the wild, both external and internal exposures are expected for much longer periods of time throughout the entire life span of the butterfly. Importantly, the present results are reminiscent of those found in previous exposure experiments [34,35,36,52,53,54,55,56] and may also explain the spatiotemporal dynamics of the abnormality rates and collection efficiency (an indicator of population density) in 2011–2013 in Fukushima [37]. Therefore, we conclude that lauric acid acts as a potent toxicant (larvicide) for the pale grass blue butterfly not only in the laboratory but also in wide polluted areas in Fukushima in the field.

This conclusion is consistent with previous studies on lauric acid as a plant defense chemical [68,69,70,71,72,73,74]. More tolerance may evolve in larvae in the field, and this scenario may explain the adaptive evolution of the butterfly shown in the polluted areas in Fukushima [56]. Interestingly, lauric acid has been reported to be a feeding stimulant for the silkworm at the concentration of 0.013% in an artificial diet [94]. This percentage corresponds to the 0.1 mg/g level in the present study. In lepidopteran insects, a feeding stimulant for a given species is often toxic to other organisms [97]. Thus, it is reasonable that a feeding stimulant for the silkworm moth, lauric acid, is toxic to the pale grass blue butterfly. Conversely, a feeding stimulant for the pale grass blue butterfly, oxalic acid [92], is probably toxic to other insects including the silkworm moth.

Alfuzosin was also demonstrated to be toxic, but its toxicity was not linearly dose dependent in the metamorphosis rates. We do not understand this nonlinearity, but it might have originated from a technical reason regarding low solubility; alfuzosin might not have been dissolved well in the diet at the relatively high concentrations. Surprisingly, in addition to the reduced metamorphosis rates, alfuzosin appeared to act on the egg-larval period to accelerate growth and tended to increase the forewing size. These results are in sharp contrast to those of lauric acid, indicating different toxic pathways in these two compounds. Because alfuzosin is an antagonist of the α_1_-adrenergic receptor [75,76,77,78], it may act on insect receptors for biogenic amines, such as octopamine and tyramine [98]. Nonetheless, both alfuzosin and lauric acid seem to affect the larval period but not the pupal period.

Because the alfuzosin concentrations tested in AD-FSW-135 were much higher than those in leaves and because the biological effects of alfuzosin and its related compound are not necessarily similar, direct extrapolations of the alfuzosin results to the alfuzosin-related compound were difficult. However, there may be a possibility that the alfuzosin-related compound was as toxic as alfuzosin due to their structural similarities. If so, the alfuzosin-related compound is 15 times as toxic as lauric acid (based on the TD_50_ values) and 12 times as lethal as lauric acid (based on the LD_50_ values), but the concentration of the alfuzosin-related compound in leaves was much lower than that of lauric acid. Therefore, the presence of the alfuzosin-related compound in leaves would not affect larvae in the field.

In contrast, ikarugamycin showed mild drug efficacy instead of toxicity. This may be simply because it is an antibiotic that inhibits bacterial or fungal growth in the artificial diet, although some antibiotics were contained in Silk Mate L4M, a commercially available ingredient of AD-FSW-135. In that case, ikarugamycin may protect the plant in the wild from fungal and bacterial infection. However, this drug efficacy of ikarugamycin for larvae may not be evident in the field because of the low concentration of ikarugamycin in leaves. Therefore, ikarugamycin would not nullify the toxicity of plant larvicides, such as lauric acid, in the field. Importantly, the present results of ikarugamycin suggest a possible contribution of metabolites from endophytic bacteria to plant and larval immunity under radiation stress. Practically, further addition of ikarugamycin or other antibiotics into AD-FSW-135 may improve its performance in the future.

In reality, in the wild, lauric acid and other upregulated unknown metabolites probably function together to ward off insects. Indeed, in response to radiation exposure, 24 upregulated peaks (*p* < 0.05) were obtained in LC–MS, among which only two of them (alfuzosin and ikarugamycin) were annotated singularly [49]. Only one upregulated peak (*p* < 0.05) was obtained in targeted GC–MS, which was lauric acid [49]. Additionally, 10 upregulated peaks (*p* < 0.05) were obtained in nontargeted GC–MS [49].

It is not possible, at least at this point, to demonstrate collective effects of many upregulated compounds with an artificial diet containing them. On the other hand, the “collective effects” have already been known by internal exposure experiments using contaminated leaves collected from Fukushima, resulting in lower survivorship and growth retardation [34,35,36,52,53,54,55,56]. We also have evidence that external exposure resulted in similar outcomes [34]. Therefore, the present finding that at least one upregulated metabolite, lauric acid, is larvicidal, is important. It is reasonable to conclude that the intricate balance between the plant and the larva through chemical interactions was affected by the Fukushima nuclear accident.

In addition to revealing the importance of plant-insect interactions in evaluating the biological effects of the Fukushima nuclear accident, this study opened new perspectives. Because ikarugamycin is likely produced by an endophytic bacterium, bacterial, fungal, or other microbial communities in plants and soil may play a role in amplifying the biological effects of low-dose radiation pollution.

## 5. Conclusions

We demonstrated within a reasonable concentration range (0.01 mg/g to 1 mg/g) that lauric acid is able to function as a toxicant for the pale grass blue butterfly at the leaf concentration (0.063 mg/g with radiation) by lowering metamorphosis rates and by causing growth retardation. Based on its TD_50_ and LD_50_ values (1.2 mg/g and 1.6 mg/g, respectively), lauric acid may be considered a mild larvicide. In the field, lauric acid probably acts as one of the larvicides in leaves in response to radiation exposure. Interpretations of alfuzosin results are not straightforward, but its relatively low TD_50_ and LD_50_ values (0.079 mg/g and 0.13 mg/g, respectively) imply that the alfuzosin-related compound may also be toxic, although it may be irrelevant in the field because of its low leaf concentration (1.6 ng/g with radiation). Because ikarugamycin is an antibiotic likely from endophytic bacteria, its drug efficacy on increasing the metamorphosis rates of larvae may be secondary; it may function to prevent the artificial diet from fungal and bacterial growth. As an extrapolation, ikarugamycin may function to protect leaves from fungi and bacteria under radiation stress. The case of ikarugamycin suggests a contribution of endophytic bacteria to the process of radiation-stress management in the plant.

In conclusion, the present results provide experimental evidence for the field effect hypothesis that concentration changes in radiation-induced metabolites, such as lauric acid, in the host plant leaves of the pale grass blue butterfly caused deterioration of the butterfly at the individual and population levels in radioactively polluted areas in Fukushima.

## Figures and Tables

**Table 1:** Concentrations of the three metabolites of interest.

Metabolite	Leaf (w/o Radiation)	Leaf (with Radiation) *^1^	Basal Level in AD-FSW-135	Toxicity Test in AD-FSW-135 *^2^	Coverage *^3^	TD_50_	LD_50_
Lauric acid	0.050 mg/g	0.063 mg/g	0.0055 mg/g	0, 0.01, 0.1, 1 mg/g	Yes	1.2 mg/g	1.6 mg/g
Alfuzosin *^4^	0.40 ng/g	1.6 ng/g	0.044 ng/g	0, 0.01, 0.1 mg/g	No	0.079 mg/g	0.13 mg/g
Ikarugamycin	0.20 ng/g	0.26 ng/g	0.022 ng/g	0, 0.01 mg/g	No	NA	NA

*^1^: For irradiation conditions, see Sakauchi et al. (2021) [49]. *^2^: Concentrations in toxicity tests ignore the basal levels of these metabolites from leaves in AD-FSW-135. *^3^: Coverage indicates if leaf concentration was covered by the tested concentration range. *^4^: Alfuzosin was tested, but the leaf concentrations shown here are those of the alfuzosin-related compound. NA: Not applicable.

## Data Availability

The data presented in this study and the source data are available in this article and in the Supplementary Materials.

## Supplementary Materials

The following file is available online at https://www.mdpi.com/article/10.3390/life12050615/s1, Table S1: Toxicological Output Data.

## Appendix A

To estimate the concentrations of the three metabolites of interest, lauric acid, alfuzosin-related compound, and ikarugamycin in leaves, the peak area values for these metabolites and oxalic acid obtained in the previous GC–MS and LC–MS analyses were compared (Figure A1). Exact values are mentioned in the Section 2. For the alfuzosin-related compound, LC–MS analyses were newly performed in the present study in triplicate to estimate its concentration in leaves using an alfuzosin standard (Sigma–Aldrich) as a reference material (Figure A2). Leaf samples used for Figure A1 and Figure A2 were identical.

We used a Shimadzu Prominence UFLC XR System (Kyoto, Japan) equipped with a Shimadzu solvent delivery unit LC-20ADXR and a Shimadzu autosampler SIL-20ACXR using a reverse-phase column Inertsil ODS-4 (2.1 mm × 150 mm) (GL Sciences, Tokyo, Japan). Mobile phase A was a 0.1% aqueous solution of formic acid, and mobile phase B was acetonitrile with a time program of its concentrations as follows: 20% (0 min) → 40% (10 min) → 98% (10.01–15 min) → 20% (15.01–23 min). The injection volume was 10 μL, and the flow rate was 0.2 mL/min.

A peak of the alfuzosin-related compound was obtained at 6.7 min from the leaf extract (Figure A2a). The alfuzosin standard also showed a peak at 6.7 min but with an additional peak at 2.1 min (Figure A2b). The latter peak was an impurity from methanol (Figure A2c). MS/MS analyses were also performed simultaneously to confirm the identities of these compounds (not shown).

## Appendix B

The exact numbers and percentages of individuals obtained after the feeding experiments are shown below for leaf and AD-FSW-135 (without any test additive) controls (Table A1), lauric acid (Table A2), alfuzosin (Table A3), and ikarugamycin (Table A4). Normalized eclosion rates (survival rates) (Table A5) and normalized normality rates (Table A6), used for Figure 5, are also shown. Further information can be found in Table S1.

**Table A1:** Number of individuals after rearing with live leaves or AD-FSW-135.

Number	Leaf	AD-FSW-135
Number of starting individuals	185 (100%)	205 (100%)
Number of pupae (Pupation rate)	168 (90.8%)	165 (80.5%)
Number of eclosion (Eclosion rate)	166 (89.7%)	141 (68.7%)
Number of normal adults (Normality rate)	156 (84.4%)	99 (48.3%)

**Table A2:** Number of individuals after oral administration of lauric acid.

Number	0 mg/g	0.01 mg/g	0.1 mg/g	1 mg/g
Number of starting individuals	75 (100%)	50 (100%)	75 (100%)	75 (100%)
Number of pupae (Pupation rate)	66 (88.0%)	41 (82.0%)	68 (90.7%)	52 (69.3%)
Number of eclosion (Eclosion rate)	56 (74.7%)	30 (60.0%)	50 (66.7%)	37 (49.3%)
Number of normal adults (Normality rate)	41 (54.7%)	19 (38.0%)	27 (36%)	23 (30.7%)

**Table A3:** Number of individuals after oral administration of alfuzosin.

Number	0 mg/g	0.01 mg/g	0.1 mg/g
Number of starting larvae	60 (100%)	60 (100%)	60 (100%)
Number of pupae (Pupation rate)	46 (76.7%)	17 (28.3%)	26 (43.3%)
Number of eclosion (Eclosion rate)	38 (63.3%)	11 (18.3%)	22 (36.7%)
Number of normal adults (Normality rate)	30 (50.0%)	7 (11.7%)	15 (25.0%)

**Table A4:** Number of individuals after oral administration of ikarugamycin.

Number	0 mg/g	0.01 mg/g
Number of starting individuals	70 (100%)	70 (100%)
Number of pupae (Pupation rate)	53 (75.7%)	62 (88.6%)
Number of eclosion (Eclosion rate)	47 (67.1%)	59 (84.3%)
Number of normal adults (Normality rate)	28 (40.0%)	35 (50.0%)

**Table A5:** Normalized eclosion rates (survival rates).

Metabolite	0 mg/g	0.01 mg/g	0.1 mg/g	1 mg/g
Lauric acid	100%	80.3%	89.3%	66.0%
Alfuzosin	100%	28.9%	58.0%	NA
Ikarugamycin	100%	125.6%	NA	NA

NA: not applicable.

**Table A6:** Normalized normality rates.

Metabolite	0 mg/g	0.01 mg/g	0.1 mg/g	1 mg/g
Lauric acid	100%	69.5%	65.8%	56.1%
Alfuzosin	100%	23.4%	50.0%	NA
Ikarugamycin	100%	125.0%	NA	NA

NA: not applicable.
